# Improving the participation of adults with visual and severe or profound intellectual disabilities: a process evaluation of a new intervention

**DOI:** 10.1186/s12913-020-05161-1

**Published:** 2020-04-16

**Authors:** Gineke Hanzen, Ruth M. A. van Nispen, Carla Vlaskamp, Eliza L. Korevaar, Aly Waninge, Annette A. J. van der Putten

**Affiliations:** 1 Royal Dutch Visio - de Brink, Groningerstraat 15, Vries the Netherlands; 2 Faculty of Behavioural and Social Sciences, department of Pedagogy and Educational Sciences, unit of special needs education and youth care, University of Groningen, Grote Rozenstraat 38, Groningen, the Netherlands; 3 Amsterdam UMC, Vrije Universiteit Amsterdam, Ophthalmology, Amsterdam Public Health research institute de Boelelaan 1117, Amsterdam, the Netherlands; 4 Hanze University of Applied Sciences Groningen, Zernikeplein 23, Groningen, the Netherlands; 5 Research group Healthy Ageing, Allied Health Care and Nursing, Hanzehogeschool Groningen, Petrus Driessenstraat 3, Groningen, the Netherlands

**Keywords:** Participation, Severe or profound intellectual disabilities, Visual disability, Development of intervention, Implementation, Process evaluation

## Abstract

**Background:**

While the participation of adults with visual and severe or profound intellectual disabilities (VSPID) in society and community life is important, evidence-based interventions to improve their participation are lacking. We conducted a process evaluation of the implementation of ‘Care for Participation+’ (CFP+), a new intervention targeting the attitudes of direct support professionals (DSPs) toward the participation of adults with VSPID, within a residential facility in the Netherlands.

**Methods:**

CFP+ was inspired by the Boston Psychiatric Rehabilitation Approach and adapted by adopting a new definition and operationalization of the concept of participation for adults with VSPID. Following systematic training, 16 DSPs of adults with VSPID were able to apply key elements of CFP+ to explore diverse roles and activities for this population, facilitating their self-management, teaching them necessary skills for participation, and organizing support. Our process evaluation entailed an investigation of the delivered dose, reach, fidelity, and adaptation of CFP+ during and after the CFP+ intervention. We also evaluated the mechanisms of impact and context using questionnaires, assignments, documentation, interviews, and a logbook.

**Results:**

The intended dose, reach, and fidelity relating to the implementation of CFP+ were not achieved. Despite this fact, an assessment of the mechanisms of impact indicated that assignments of CFP+ were well (75%) or reasonably well (17%) understood by DSPs. CFP+ was applied by DSPs to stimulate self-management (83% of DSPs), new activities (100%), enhanced involvement in existing activities (67%) and to explore new roles (50%) for adults with VSPID. A negative contextual factor mentioned by the trainer and manager was the DSPs’ lack of commitment to the training program. Another negative contextual factor mentioned by DSPs was the lack of time for implementing CFP+.

**Conclusions:**

CFP+ provides new opportunities to improve the participation of adults with VSPID. Despite the non-optimal conditions for implementing CFP+ and the DSPs’ general reluctance to apply the new intervention, some have actively used CFP+ within the residential facility. Future studies should focus on the outcomes of CFP+ regarding attitudinal changes among DSPs relating to the participation of adults with VSPID and their quality of life.

## Background

Individuals with severe or profound intellectual disabilities frequently also experience visual limitations as well [1]. In the Netherlands, adults with visual and severe or profound intellectual disabilities (VSPID) comprise approximately 0.05 to 0.08% of the Dutch population [2]. These adults have a visual impairment (visual acuity < 6/18) or blindness (visual acuity < 3/60 and/or visual field < 10 degrees around the point of fixation), as defined by World Health Organization criteria, and an intelligence quotient of less than 35 points [3]. In addition, they often experience other sensory impairments (e.g. hearing loss), behavior problems (e.g. challenging behavior), and health problems [4–6]. Research by Van Timmeren, Van der Putten, Van Schrojenstein Lantman-de Valk, Van der Schans, and Waninge [6] has shown that an individual with VSPID has on average 12 health problems; in more than 50% epilepsy, spasticity, constipation, incontinence, deformations, and reflux has been reported. These problems of adults with VSPID are interrelated. For example, adults with VSPID cannot compensate their intellectual disability by using vision or compensate vision loss by employing their cognitive skills. Since these compensation mechanisms are not in place, the visual and intellectual disabilities seem to reinforce each other [7], which causes additional limitations in daily activities, e.g. living skills, communication, initiative, and social skills [8, 9]. Because of all these limitations, persons with VSPID are fully dependent on others and often live in residential care facilities [4]. Their dependence on others is complicated by the fact that they often communicate non-verbally, through facial expressions, vocalization and body language [10], and therefore, it is often not clear what their needs and preferences are to direct support professionals (DSPs) and family members. Considerable knowledge is required from DSPs and family members to explain the meaning of the behavior of individuals with VSPID. The accumulation of impairments, combined with the difficulties in explaining their behavior, makes people with VSPID a vulnerable group experiencing limitations and depending on others in all aspects of their lives. As a result, interventions that have been developed for people with intellectual disabilities are generally not suitable for individuals with VSPID because these interventions do not take sufficient account of the many and complex problems of the target group.

The United Nations Convention on the Rights of People with Disabilities [11] provides adults with VSPID with the right to participate fully in society and in community life. This Convention has been in effect in the Netherlands since July 14, 2016 [12]. Several studies have highlighted the importance of participation for individuals with severe intellectual disabilities (e.g., [13, 14]). Participation may contribute to an individual’s development and emotional well-being [15, 16], as well as to better quality of life [17]. Due to the complex and interrelated limitations of adults with VSPID, it is a major challenge for DSPs to operationalize a broad concept such as participation. Consequently, a specific definition and operationalization of the concept of participation in relation to these individuals was necessary and, formulated in former research as follows:

*Active engagement and involvement in daily activities, social contacts, and societal and leisure activities, including opportunities for inclusion, experiences, and discovery. Active engagement and involvement of this population can only occur in the context of a relationship with the environment (‘being understood’) wherein the adult with VSPID has an active and steering role (‘self-management and autonomy’)* [18].

The concept and operationalization of participation for adults with VSPID is relatively new and has not yet become established within society. A recent study of Hanzen, Waninge, Vlaskamp, Van Nispen, and Van der Putten [19] within residential facilities revealed that the support offered by DSPs in terms of participation appeared to focus mainly on having or maintaining social relations, gaining sensory experiences, and engaging in (daily) activities that matched their interests. Their participation was found to be much less focused on finding new leisure activities and seeking inclusion within society, especially outside of the residential facility. In addition, no efforts were made to change or introduce new social roles for adults with VSPID that could enhance their participation. These findings are in line with the results of a study of Talman, Gustafsson, Stier, and Wilder [20], which also showed that support professionals find it difficult to define potential roles for adults with profound intellectual (and multiple) disabilities. The importance of social roles have previously been described by Wolfensberger [21]. Related to the limitations of individuals with VSPID, frequently described examples of roles of people with VSPID are: ‘client’, ‘patient’ or ‘participant of daycare activities’. Becoming aware of other (active) roles individuals with VSPID already have, such as ‘a son’ or ‘a neighbor’, or new roles they could have, such as e.g. ‘an animal caretaker’ (filled with activities such as stroking and helping to feed a rabbit), ‘an assistant cook’ (with an activity such as pressing a button to operate the mixer) instead of ‘client’, could encourage residential care facilities to develop more active and more suitable activities for the individual with VSPID. Because adults with VSPID are highly dependent on their environment and the support they receive from others [4], a possible explanation for their limited participation could lie in the attitudes and resources of DSPs. Research has shown that DSPs find it difficult to apply inclusive principles, which are key components of participation, in relation to individuals with severe or profound intellectual disabilities [22, 23]. Maxell and colleagues [24] concluded that other environmental factors, such as the availability of facilities or resources, accessibility to a specific situation, and affordability (financial constraints) may also result in limited participation.

In order to achieve a satisfactory level of participation of adults with VSPID within society and community life, new requirements have been imposed on society, including its residential facilities. As this is a relatively new development in the Netherlands, residential facilities have been actively seeking appropriate interventions for enhancing the participation of adults with VSPID [19]. Despite the implementation of initiatives to increase the participation of individuals with intellectual disabilities [25], until now, training for DSPs in residential facilities has mainly focused narrowly on their role as caretakers. Consequently, and especially in residential facilities, DSPs prioritize support relating to the provision of care and devote less attention to the issue of societal inclusion [26].

A number of interventions have been developed that appear to address only specific components of participation for adults with VSPID, as operationalized by Hanzen et al. [18]. For example, an intervention to improve community inclusion, described by Bolsenbroek [27], aims for an inclusive society for people with disabilities and uses insights from social role valorization. Interventions to increase engagement in social networks are described by Kruijswijk and colleagues [28]; these interventions are primarily aimed at people with mild or moderate intellectual disabilities. Another component of participation, self-management, is the aim of an intervention called ‘On Your Own Two Feet’ [29]. This intervention teaches support staff to encourage persons with intellectual disabilities to think about and solve problems by themselves, which could improve their self-management: due to the limitations in intellectual capacity, this intervention is not applicable for individuals with VSPID. In addition, an intervention termed “active support” has been developed for adults with intellectual disabilities aimed at strengthening their engagement in daily activities with appropriate staff support [30, 31]. The Boston Psychiatric Rehabilitation Approach (BPRA), entailing a broad approach to participation, was introduced in the Netherlands in 1992 [32]. This intervention, which was developed by the Center for Psychiatric Rehabilitation in Boston, supports individuals with psychiatric disabilities in achieving their participation needs [33]. However, the BPRA is less suitable for individuals with VSPID because it requires conversational skills that such individuals do not possess.

In sum, appropriate interventions for adults with VSPID as well as broader ones encompassing the participation areas ‘to experience and discover’, ‘inclusion’, ‘involvement’, ‘leisure and recreation’, ‘communication and being understood’, ‘social relations’, and ‘self-management and autonomy’, as described by Hanzen et al. [18], are lacking. Therefore, we developed an intervention, called ‘Care for Participation+’ (CFP+), designed to increase the participation of adults with VSPID. Since any implementation process affects the potential effects of an intervention, it is important to evaluate the implementation by a process evaluation [34–36]. Thus, the aim of this study was to conduct a process evaluation to observe the implementation phase of CFP+ within a residential facility.

## Methods

### Design

We conducted a process evaluation of the CFP+ intervention using measurements during the implementation phase. The intervention targeted one group of DSPs and adults with VSPID within a residential facility for people with VSPID in the Netherlands. Data were collected prior to implementation of the CFP+ intervention. In addition, measurements as described in Table 1 were taken during the training sessions, immediately after the conclusion of the training sessions, and four and six months after the intervention’s implementation (see Table 1).

**Table 1:** Operationalization of variables and data collection

Variable	Data source	Time of data collection^a^
**Implementation process, dose, adaptation, fidelity, and reach**		
Implementation process: -Information sent in advance to the management of the residential facility -Information sent in advance to DSPs -Arrangements made within the residential facility	Logbook of researcher (GH)	T0
Characteristics of the DSPs	Online questionnaire completed by DSPs	T0
Adaptation of CFP+ during training	Logbook of researcher (GH)	T1
Dose: DSPs who received CFP+ training	Logbook of researcher (GH)	T1, T2
Dose of CFP+ training	Logbook of researcher (GH)	T1, T2
Dose: Time spent by DSPs practicing CFP+ after the training	Online questionnaire completed by DSPs	T3
Fidelity: conducting assignments	Evaluation forms completed by DSPs	T2
Fidelity: Use of worksheets by the DSPs as part of the intervention	Worksheets completed by DSPs	T1
	Online questionnaire completed by DSPs	T3
Fidelity: Extension of CFP+ to self-management, new activities, and greater involvement in existing activities of adults with VSPID	Telephone interviews conducted with DSPs	T4
Reach: dissemination of CFP+ by DSPs among team members	Telephone interviews conducted with DSPs	T4
**Mechanism operating during the intervention that could have influenced the outcomes**		
Quality of the teaching imparted by the trainer, as perceived by DSPs	Evaluation forms completed by DSPs	T2
Applicability of the teaching material as perceived by DSPs	Evaluation forms completed by DSPs	T2
Relevance for the work of DSPs	Evaluation forms completed by DSPs	T2
DSPs’ understanding of the assignments in the worksheets	Worksheets completed by DSPs	T1
DSPs’ logical choices reflected in successive worksheets	Worksheets completed by DSPs	T1
Trainer’s feedback on the training of DSPs and their use of CFP+ tools	Evaluations of the trainer and one of the CFP+ developers	T2
Trainer’s feedback on the behavior of the group during the training period	Evaluations of the trainer and one of the CFP+ developers	T2
Manager’s feedback on the behavior of the group during the training period	Evaluation interview conducted with the manager of the residential facility	T2
**Contextual factors that could have affected CFP+ outcomes**		
DSPs’ feedback on positive and negative conditions relating to the implementation of the CFP+ intervention	Evaluation forms completed by DSPs	T2
Trainer’s feedback on the positive and negative conditions relating to the implementation of the CFP+ intervention	Evaluations of the trainer and one of the CFP+ developers	T2
Manager’s feedback on positive and negative conditions relating to the implementation of the CFP+ intervention	Evaluation interview conducted with the manager of the residential facility	T2

*DSP* direct support professional, *CFP+* Care for Participation+,^a^*T0* before training, *T1* during training, *T2* after training, *T3* 4 months after training, *T4* 6 months after training

### Development of the CFP+ intervention

#### Preliminary version of the CFP+ intervention

During an earlier phase of our work, the management and DSPs of a residential facility for people with VSPID indicated that they would like to promote the participation of their target group; the family of the people with VSPID also supported this goal. Since no suitable intervention was available for the target group, we developed Care for Participation (CFP) as a preliminary intervention for increasing the participation of adults with VSPID [37] (see ‘Content of CFP+’). CFP was initially implemented through the delivery of a training program for DSPs who worked with adults with VSPID. CFP was inspired by the BPRA intervention that is designed to enhance the participation of individuals with psychiatric disabilities [33]. There are several reasons why the BPRA was chosen as the basis for CFP. The first relates to the BPRA’s core underlying assumption that individuals have wishes, needs, and strengths rather than problems and limitations. Because adults with VSPID have many disabilities, their limitations rather than their strengths are often the focus of attention. This is in line with research conducted by Bigby et al. [22] which indicated that the behavior of most support professionals towards inclusion is based on the attitude that the principles of inclusion and participation were not applicable for individuals with severe or profound intellectual disabilities. In addition, Talman et al. [23] showed that participation of individuals with profound and multiple disabilities was often reduced because support professionals believed these people were not capable of participation. Therefore, a new intervention should also focus on improving the attitudes of DSPs regarding the participation of adults with VSPID. According to Pickens [38], changing an individual’s attitude requires a focus on its three components: an affect (feeling), cognition (a belief or thought), and behavior (an action).

A second reason why we based our intervention on the BPRA relates to its aim of improving the various life roles of an individual, such as those of a son, an employee, or a friend. Adults with VSPID often have fewer roles than other individuals and those that they have mostly entail just a few activities [19].

A final set of reasons for the choice of the BPRA relates to the fact that it has a systematic structure, is easily transferable, and has proven to be effective when applied to the target group for which it was designed [39, 40]. The BPRA is a tailor-made, and context-free approach and can therefore be applied to multiple target groups. However, because the BPRA is framed as a conversational model, it needed to be adjusted for the population of adults with VSPID who have limited or no possibilities of language-based speech [37].

The BPRA was therefore modified and applied in a residential facility for individuals with VSPID. In this facility, the topic of participation and the possibilities offered by the BPRA approach were introduced to the managers, families, and DSPs of individuals with VSPID. The positive reactions to the BPRA’s vision and systematic method led to the adjustment of the BPRA to make it appropriate for adults with VSPID. A BPRA specialist and an expert on adults with VSPID (the first author) jointly developed the CFP intervention, which included a four-day training program for DSPs working with adults with VSPID. The BPRA principles such as emphasis on wishes, needs, and strengths and on role functioning were retained in this modified intervention, but the method was changed from a conversation-oriented method to one that could be used in daily practice relating to the target population. The involvement of colleagues and the families of adults with VSPID enabled the DSPs to deploy their newly acquired skills to develop the roles and activities of adults with VSPID.

The CFP intervention was tested in a pilot study conducted at the same residential facility for individuals with VSPID [37]. The selection of DSPs was a convenience sample. The selected DSPs were asked if they were interested in the subject and if they liked to participate. During and after the training CFP, the 12 selected DSPs contributed to the further development of CFP by assessing which aspects of the CFP approach could be practically applied and which aspects required adaptation.

The results of the pilot study, obtained by analysis of questionnaires, files, and a logbook, indicated that the CFP approach could be applied for adults with VSPID. As a result of their use of the approach, DSPs were more focused on the strengths of adults with VSPID than on their disabilities. Moreover, the range of activities in which adults with VSPID engaged in daily practice had increased. DSPs noted the importance of integrating the CFP approach within the workflow and in case deliberations to strengthen its applicability. In addition, time was allocated for conducting evaluations of the CFP components. Because these evaluations were not required for the follow-up training, the duration of this training program was reduced from four to three days. Furthermore, the recommendations made during the pilot study were to focus on the concept of participation of adults with VSPID and on the applicability and long-term effects of CFP within larger groups of DSPs and adults with VSPID.

#### Adaptation of CFP and the development of CFP+

After consulting experts on BPRA and VSPID, the second stage of developing the CFP approach was initiated that retained the essential aspects of the CFP approach while integrating the definition and operationalization of the concept of participation within the intervention [18]. The definition and operationalization of participation were developed from the perspectives of proxies of the adults with VSPID using an online concept mapping procedure. This process led to the creation of a Participation Mind Map (PMM) that explains the definition and provides practical examples covering the seven areas described by Hanzen et al. [18]: experience and discover, inclusion, involvement, leisure and recreation, communication and being understood, social relations, and self-management and autonomy.

The PMM was integrated into the CFP+ approach in multiple ways. First, the PMM was included in the training material to enable its use during the initial steps of the CFP+ implementation process, entailing an exploration of the wishes and strengths of adults with VSPID. Second, elements of the PMM were added to the mission statement as well as to the initial and concluding (evaluation) sections of the CFP+ manual. Third, specific exercises for the DSPs, and goals associated with the achievement of more autonomy and more active involvement of adults with VSPID, were added to CFP+ to be incorporated into daily practice.

#### Content of CFP+

CFP+ is taught systematically with the aid of a manual including a training overview, the PMM, and worksheets with exercises covering each step of the CFP+ process. CFP+ comprises the following seven steps:

1. *Exploring opportunities for increasing the self-management and autonomy of an adult with VSPID*

The roles of an adult with VSPID and the activities through which those roles can be fulfilled are entered into a pre-established scheme. This scheme is then compared with the concerned person’s personal profile that has been recorded by the DSP and by other significant individuals, providing details on the person’s character, preferences, and strengths. The scheme should match the above-described personal profile. Possible outcomes entail a complete or partial fit of the activities with the profile or no fit at all.

2. *Exploring possible areas of dissatisfaction and hidden needs/wishes*

The DSP observes any dissatisfaction displayed by the adult with VSPID which could signify the need for a change and for the exploration of new activities or the elimination of obstacles to restore the individual’s satisfaction with existing activities.

3. *Choosing and formulating a goal: Developing a new activity and/or strengthening involvement in an existing activity*

Possible wishes of the adult with VSPID are translated into a goal that is discussed by the DSP with the family and with colleagues. In a process that is as creative as possible, the DSP then comes up with new eligible activities, striving to be fully open and discounting any limitations, circumstances, or conditions relating to the concerned individual. Considering the individual’s preferences, the DSP sets a goal that precisely describes the role, activity, and preferred environment of the adult with VSPID.

4. *Achieving the goal*

In consultation with colleagues and with the individual’s family, the DSP determines what needs to be done to achieve the goal. During a brainstorming session, the DSPs are asked to think about factors that could contribute to making the goal attainable. Following this session, the various factors are listed under the heading of skills recorded for the adult with VSPID and under the heading of required support that can be obtained within the environment of the intervention. DSPs are taught to assess whether the goal enhances the satisfaction of the adult with VSPID as well as the satisfaction of those within the person’s environment.

5. *Teaching necessary skills to an adult with VSPID*

If an adult with VSPID needs to learn new skills to achieve a goal, the DSP considers whether or not the partial imparting of these skills to the individual is feasible. Through skills development, the individual’s autonomy and self-management can be increased. If it is not possible to impart the required skills, the DSP will determine whether the goal can be achieved with support available within the environment.

6. *Organizing support*

The DSP will assess the type of support required to enable the adult with VSPID to carry out certain activities. This support may comprise resources and appointments with colleagues, volunteers, or family members who want to help the concerned individual to perform the desired activities. The DSP is trained to present the strengths and positive aspects of an adult with VSPID to the network of individuals who can offer support, considering their motivations and expectations.

7. *Problem solving*

The DSP is trained to evaluate new activities by describing the signs and gestures of an adult with VSPID that indicate involvement in and pleasure derived from an activity. Furthermore, the DSPs are trained to investigate factors that hinder the performance of activities and to design and implement an appropriate solution in a systematic manner.

**Fig. 1:**
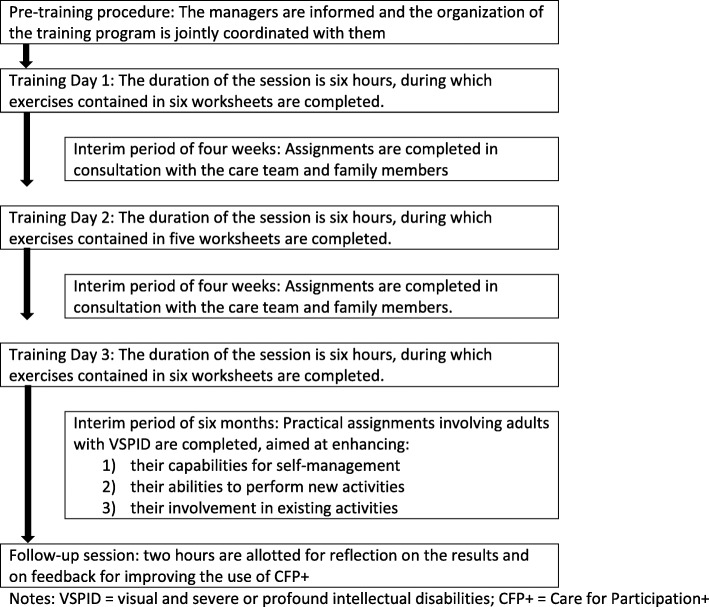
Planned schedule for the implementation of the CFP+ intervention. Notes: VSPID = visual and severe or profound intellectual disabilities; CFP+ = Care for Participation+

Figure 1 depicts the schedule for the implementation of the final version of the CFP+ intervention. The time lapse between consecutive days of training organized for the DSPs of adults with VSPID (three in total) was about four weeks. During the training program, the DSPs conducted exercises that could feasibly be performed as part of their daily practice. During the interim periods, the DSPs completed assignments involving their colleagues and the family members of the adults with VSPID. This involvement was deemed necessary for acquiring a better understanding of adults with VSPID and were considered prerequisites for improving participation. Six months after the training program concluded, a two-hour session was held during which the DSPs reflected on the results of CFP+ and the problems they had encountered when attempting to execute the goals they had formulated. In consultation with the trainer, the DSPs searched for “anchors” within the CFP+ intervention that they could use to solve specific problems. To ensure that DSPs continue to use the CFP+ approach in future, this two-hour session program should be held on an annual basis.

### Participants

A total of 16 DSPs participated in the process evaluation conducted during and after the delivery of the CFP+. Regarding selection of participants for CFP+, management decided within which homes of the residential facility DSPs were asked to participate in the study. The CFP+ trainer had set the maximum number of participants at 16: this way there was enough time to give every DSP sufficient attention and guidance during training.

In addition to providing the usual support for the individuals they worked with, DSPs received training and implemented the CFP+ intervention. The inclusion criteria of the DSPs were that they had at least six months of experience in supervising adults with VSPID in their homes at the residential facility or during daytime activities at the residential facility, and had expressed their intentions of continuing to support the adults with VSPID assigned to them throughout the study period. Each DSP was linked with an adult with VSPID with whom the DSP usually worked. Inclusion criteria for adults with VSPID were that they were at least 21 years old, had a visual impairment (visual acuity < 6/18 and/or visual field < 20 degrees around the point of fixation) or blindness (visual acuity < 3/60 and/or visual field < 10 degrees around the point of fixation) [41], and an intelligence quotient of less than 35 points. Additional chronic (health) problems that were considered stable were not included among the exclusion criteria. These criteria included diseases with an expected prognosis of a strong decline within one year and expected organizational disturbance within the group the adult with VSPID is living.

The DSPs and family members of adults with VSPID were informed about the study and provided their informed consent in writing. The study protocol for pilot testing CFP+ was approved by the Ethical Committee of the Department of Special Needs Education and Youth Care at the University of Groningen in the Netherlands.

### Data collection

Table 1 shows the operationalization of the variables, the data sources, and the timing of data collection.

We followed the guidelines of the UK Medical Research Council when conducting the process evaluation [42]. As confirmed by Moore et al. [36], this guideline is relevant for conducting process evaluations of public health interventions as well as for complex intervention research in other areas of healthcare or in education. While variations in process evaluations are acknowledged within these guidelines, they specify three key aspects that researchers should prioritize in their investigations: implementation, mechanisms of impact, and context [36]. We operationalized and studied these aspects according to the following definitions: 1) The implementation process was experienced or defined in terms of the dose, adaptation, fidelity, and reach of CFP+ in practice; 2) Mechanisms of impact referred to those mechanisms generated by the CFP+ intervention that could have triggered changes in outcomes in terms of the DSPs’ responses and potential mediators; and 3) Context referred to positive as well as negative contextual factors that affected the CFP+ intervention, as experienced by DSPs.

Data were gathered from DSPs who had received CFP+ training for the process evaluation. Additional observations were obtained from the trainer and the manager who supervised the study within the residential facility. Data were gathered before, during, and at the conclusion of the training program. Additional data were gathered four and six months after the conclusion of the training program (see supplementary file).

### Data analyses

Documentary and content analyses were performed on qualitative data (see Table 1) while descriptive statistics were applied in the analysis of quantitative data (see Table 1) extracted from the evaluation forms. Included in the documentary and content analyses were (1) the notes in the logbook with regard to the information provided to management and DSPs in advance, the dosage, and adaptation of CFP+ were included in the analysis; (2) the worksheets of the DSPs, completed during the training, have been analyzed to evaluate whether the DSPs had properly understood the assignments and CFP+ in general. Using a four point Likert scale, it was assessed whether or not the DSPs understood the worksheets.(3) the structured interviews, they have been audio-recorded, and the answers to the predetermined questions about the implementation of CFP+ have been included in the analysis.

## Results

Following the guidelines of the UK Medical Research Council, the results of the process evaluation of the implementation of CFP+ are organized in three chapters: (1) the implementation of CFP+ in practice which describes the implementation process, the dose, adaptation, fidelity, and reach; (2) Mechanism during the implementation period of CFP+ that could have influenced the outcomes; and (3) Contextual factors, either positive or negative, that may have affected the implementation of the intervention. Table 2 presents a summary of the findings of the process evaluation of the CFP+ intervention.

**Table 2:** Findings of the process evaluation

**Implementation process, adaptation, dose, fidelity, reach**	
Implementation:	
-Information conveyed to management	May 2017
-Information conveyed to the DSPs	June 2017
-Arrangements made within the residential facility	September–November 2017
Adaptation of the CFP+ during training	Two components were added during the training: Explanations provided by the coordinator of the volunteers Demonstration of new activities developed for adults with VSPID
Dose: Number of DSPs who received CFP+ training	14/16 present on the first day of training 13/16 present on second day of training 14/16 present on the third day of training
Dose: Intended training time	*N* = 12 h: 66.7% of intended training time Feedback session replaced by telephone contact
Dose: Time spent practicing CFP+ during the interval between the completion of training and T2^b^	N = 8^a^: 1 DSP spent 40 min, 1 DSP spent 30 min, 6 DSPs spent 0 min; Eight missing values
Fidelity: carrying out assignments during the training program, as reported by DSPs	N = 1: 7.1%: good *N* = 6: 42.9%: neutral *N* = 5: 35.7%: moderate *N* = 2: 14.3%: insufficient Two missing values
Fidelity: Use of the worksheets during and after the training	During the training: *n* = 169: 88% completed T2^b^: 0% completed (not used)
Fidelity: Concrete application of CFP+ up to T2, as reported by DSPs in the areas of:	12/16 reported
-self-management for adults with VSPID	N = 10: 83.3%
-new activities for adults with VSPID	N = 12: 100%
-involvement of adults with VSPID in existing activities	N = 8: 66.7%
-new roles for adults with VSPID	N = 6: 50%
Reach: dissemination of the CFP+ by DSPs within the team up to T2	*N* = 3: 25%: no dissemination *N* = 9: 75%: partial dissemination Four missing values
**Mechanisms generated by CFP+ itself that could have influenced the outcomes: DSPs responses and mediators**	
Quality of trainer’s teaching, as reported by DSPs just after the training program	N = 7: 43.8%: good N = 8: 50%: neutral *N* = 1: 3.1%: moderate One missing value
Practical applicability of teaching material, as reported by DSPs just after the training program	N = 4: 26.7%: good N = 2: 13.3%: neutral *N* = 8: 53.3%: moderate N = 1: 6.7%: insufficient One missing value
Appropriateness in relation to DSPs’ work, as reported by DSPs just after the training program	N = 1: 6.3%: good *N* = 2: 12.5%: neutral *N* = 6: 37.5%: moderate *N* = 7: 43.8%: insufficient
Understanding of the assignments provided in the worksheets during the training sessions, as assessed by the researcher (GH)	*N* = 129: 74.6%: well understood *N* = 29: 17.2%: reasonably understood *N* = 10: 5.9%: moderately understood *N* = 4: 2.4%: insufficiently understood
Responses in successive worksheets completed during the training sessions reflect logical choices, as assessed by the researcher (GH)	N = 29: 45.3%: satisfactory logical sequence N = 7: 10.9%: reasonably logical sequence N = 6: 9.3%: moderate logical sequence *N* = 5: 7.8%: absence of a logical sequence 17 missing values
Trainer’s feedback about the training group and the DSPs’ use of the CFP+ during the training program	Difficult to foster self-reflection Not aware of the added value of CFP+
Trainer’s feedback regarding the behavior of the group during the training program	Not focused and poor concentration Dominance of some of the DSPs
**Contextual factors that affected the implementation of CFP+**	
DSPs’ feedback on positive and negative conditions for implementing the CFP+ intervention	Not consulted by the manager or trainer to provide inputs prior to the training Lack of time to implement CFP+ up to T2 Nonavilability of volunteers for implementing new activities up to T2
Trainer’s feedback on positive and negative conditions for implementing the CFP+ intervention	Lack of commitment to the training demonstrated by DSPs
Manager’s feedback on positive and negative conditions for implementing the CFP+ intervention	Convinced of CFP + ‘s added value both before and after the training program Convinced of the existence of opportunities for implementing CFP+ before and after the training program DSPs’ lack of commitment to the training program

*DSP* direct support professional, *CFP+* Care for participation+

^a^Three DSPs could not be interviewed because they had changed jobs and were no longer employed by the residential facility; one DSP was absent; ^b^T2 = 6 months after the training

### Implementation in practice

Prior to implementing the intervention, the manager of the residential facility was informed about the purpose and content of the training program, and she subsequently informed the DSPs. The training program was scheduled to be held in a classroom within the residential facility over three days.

A total of 16 DSPs from the residential facility signed up for the CFP+ training program. Reasons for absences during the training were related to familial or work circumstances. Six months after the first training session was held, three of the DSPs in the original group had changed jobs and were no longer employed at the residential facility.

All of the DSPs were women, and their mean age was 35.6 years (ranging between 20 and 55 years). All of them belonged to the intended target group of DSPs working with adults with VSPID: nine worked as DSPs supporting in a home group at the residential facility, two worked as DSPs supporting in a daytime activity group at the residential facility, and five had a coordinating role, in addition to their supervisory roles.

The CFP+ training sessions were not carried out as scheduled; the training time had to be reduced from the planned six hours to four hours on each of the days of training because noise from the adjacent room affected the concentration spans of the trainees. In light of feasibility issues, and at the DSPs’ request, the two-hour follow-up session that was scheduled to be held six months after the last day of training was replaced by a telephone conversation with each DSP. During this session, the DSP was reminded of the utilization and possibilities of CFP+ and of the possibility of requesting assistance to advance their use of CFP+.

As shown in Table 2, DSPs indicated in their evaluation forms that they were not always able to complete the assignments that were set for the periods between the training days for several reasons. These included “too little time,” “it was not possible because the family lives far away,” or “I had already filled it in during the training session.”

During the training program, two additional components were added to the CFP+ that fitted within this specific residential facility and had a direct bearing on the possibilities for enhancing participation of the adults with VSPID. First, the individual who coordinated the volunteers at the residential facility explained the opportunities of the volunteers to the trainees (fits well in step six ‘organizing support’ of CFP+) and second, one of the DSPs introduced a number of new activities for adults with VSPID (fits well in step three ‘choosing and formulating goals’ of CFP+).

During the training program, 12 worksheets with exercises, spread over the three days training, were discussed by the DSPs with the trainer. Of the 192 worksheets (12 worksheets × 16 DSPs), a total of 169 (88%) were completed by the DSPs.

Six months after the conclusion of the training program, the DSPs reported that they had not applied the exercises in the worksheets during the post-training period as instructed in relation to the adults with VSPID with whom they were associated during the study; nor had they applied these exercises in relation to any other adult with VSPID with whom they had worked. The most frequently mentioned reason provided by the DSPs for not implementing the worksheet exercises was the overlap with two other tools that are used within the residential facility: a diagnostic questionnaire and a management tool. Out of the 16 DSPs who received training, eight mentioned that they had spent between 0 and 40 min using the CFP+ methodology during the six-month period that followed the training.

After the training program, the DSPs applied the goals of the worksheets they had completed during the training in their daily practice: they reported working in the following areas: developing new activities, self-management and autonomy, active involvement in existing activities, and new roles.

Six months after the conclusion of the training, DSPs reported that while they had not implemented the CFP+ methodology in their daily practice, they had informed their colleagues about CFP+, indicating the achievement of reach. However, some DSPs reported that they had not disseminated the intervention practices within their teams.

### Mechanisms generated by CFP+ that could have influenced the outcomes

The second aspect highlighted by the UK Medical Research Council and included in the process evaluation was mechanisms of impact, operationalized as mechanisms generated by the CFP+ intervention that could have triggered changes in outcomes in terms of DSPs’ responses and potential mediators.

The DSPs’ experiences of the CFP+ intervention and associated training program ranged from evaluations that it was “clear” to a view that it provided *“*good training but nothing new for us.*”* Other relevant comments were that “The training would be very suitable for new employees because you learn to focus on the possibilities of the population in a different way” and “cooperation with other disciplines where work has been done with adults with VSPID has added value.” In addition, there were comments about the overlap with other interventions already used within the residential facility, such as “[there is] a lot of repetition; we already do many [of these] things.”

The analysis of the worksheets revealed whether the DSPs had properly understood the assignments. For example, the DSPs had to write about the different roles of the adults with VSPID with whom they worked in one of the worksheet assignments. If they listed roles such as *“*brother*”* or “roommate,” these answers demonstrated a correspondence with the assignment, revealing that the DSP had understood its purpose. However, responses such as “he is very kind” or “he likes to swim,” revealed a lack of correspondence with the purpose of the assignment, indicating that the DSP did not understand the assignment provided in the worksheet. Of the 169 completed worksheets, 126 worksheets indicated that the assignments were well understood (almost everything that was entered on the worksheet met the requirements of the assignment), 29 indicated that the assignments were reasonably well understood (the number of statements that matched the assignment exceeded those that were not correct), 10 were moderately well understood (the number of statements that matched the assignment was less than the number of statements that were correct), and four were insufficiently understood (almost all of the contents entered on the worksheet did not tally with the requirements of the assignment).

During the training period, we also assessed the extent of the DSPs’ understanding of the cohesive nature of the CFP+ intervention. There were four occasions during the CFP+ training program when it was possible to determine whether the answers provided by the DSPs on successive worksheets matched and whether they had made logical choices. For example, in one of the worksheets, the DSPs had to formulate a goal for a new activity. In a subsequent worksheet, the DSP identified the skills that an adult with VSPID would have to acquire for conducting this new activity. An example of a goal was “be involved in cooking at home”. If the DSP noted that the adult with VSPID “has to learn to stir the contents of the pan” in the following worksheet, this was considered to be a logical choice that was appropriate for the goal described in the previous worksheet. For the 16 DSPs, there were 64 (four occasions for 16 DSPs) possible sequence results. Of these results, 17 were missing (incomplete worksheets). From the remaining results, 29 results indicated a satisfactory logical sequence (almost everything on the worksheet followed logically from the previous worksheet). There were seven reasonable logical sequences (several statements followed logically), six moderate logical sequences (some statements followed logically), and five insufficient logical sequences (almost no logical connection existed with the previous worksheet).

The trainer further indicated that it was “difficult to provoke [DSPs’] self-reflection” and that “an in-depth understanding of the curriculum was not achieved.” Moreover, she made the following observation:

From the submitted worksheets it appeared that parts were well used and could be used. However, from what the trained DSPs reported, it did not appear that they were aware of this. It is therefore to be expected that they will not include these worksheets in their repertoire of actions.

A number of the DSPs indicated that the training group was not focused. They reported “no enthusiasm and little attention,” “too much distraction and limited concentration,” and “too much distraction because the information is not new.” The trainer also confirmed that the DSPs’ concentration during the training was poor and that the opinions of some DSPs were decisive for the others:

A block was created, with a number of prominent DSPs having a decisive influence. It took a lot of effort to get others to speak. They sometimes had different ideas about the opportunities of CFP+, but did not get these across, or did not put much effort into it. The opinion that prevailed was, "we already do what is expected for CFP+ and this adds nothing new.’ Consequently, it was not possible to provoke a curious or inquiring attitude among the DSPs.

During the evaluation, the manager indicated that the DSPs did not find that the training program added much value to their work. However, according to the manager, the DSPs could hardly envisage how CFP+ was related to other approaches applied within the residential facility. She stated that a diagnostic questionnaire was in fact used, but unlike CFP+, this questionnaire did not constitute a systematic method with associated practical tools for achieving goals. In addition, the manager acknowledged the poor concentration of the DSPs during the training, revealing that this also applied to other training sessions that had been held within the residential facility. A possible explanation that she offered was that the DSPs exchanged work experiences during the training sessions because they do not have time to do so during regular working hours.

### Contextual factors that affected the implementation of CFP+ intervention

The third aspect highlighted by the UK Medical Research Council and included in the process evaluation was context, operationalized as positive and negative contextual factors that affected CFP+, as experienced by DSPs.

Positive conditions mentioned by the manager were that the management was convinced, before as well as after the training program, of the added value that CFP+ provided. Moreover, the manager felt that there were opportunities for implementing at least some components of CFP+ within the residential facility.

Negative factors mentioned by the DSPs related to the lack of time for practicing CFP+ and of available volunteers for implementing new activities for adults with VSPID. Furthermore, the DSPs indicated that, unlike the management, they were not sufficiently informed and consulted before the commencement of the training program. Both the manager and the trainer pointed to a lack of commitment to the training among the DSPs because they felt that their participation was based on a top-down decision that was “forced” on them and because they found that it overlapped with other approaches used within the residential facility.

## Discussion

The purpose of this study was to examine the implementation process of a new intervention for enhancing the participation of adults with VSPID within the daily practices at a residential facility in the Netherlands. The study described the development of the CFP+ intervention which included a training for DSPs aimed at improving the participation of adults with VSPID according to the broad perspective of participation as described by Hanzen et al. [18]. CFP+ entails a systematic method designed to change DSPs’ attitudes toward the participation of adults with VSPID. Moreover, it is aimed at helping DSPs to improve these individuals’ self-management and autonomy as well as to organize new activities for them or to increase their involvement in existing activities.

An important element of CFP+ entails its emphasis on the diverse roles that individuals with VSPID can have in different areas of life, such as social relations or leisure and recreation. This emphasis on varying roles that such individuals can assume within and outside the residential facility may induce changes in the attitudes of DSPs regarding the possibilities, opportunities, and activities that apply to the adults with VSPID with whom they work. The importance of attitudinal changes has been demonstrated in a study conducted by Talman et al. [20], who found that DSPs experience difficulty in developing new roles for individuals with profound intellectual disabilities. Experiences of implementing the preliminary version of the CFP+ intervention revealed that as a result of the intervention, DSPs focused more on possibilities and less on the disabilities of adults with VSPID. Consequently, they increased the range of activities for adults with VSPID within daily practices [37].

The findings of this process evaluation show that operationalization of the concept of participation in the context of adults with VSPID [18] closely matched that of the preliminary version of the intervention, with the inclusion of additional elements. The CFP+ intervention enabled DSPs to expand their focus to other areas of participation. Thus, in addition to developing new activities for adults with VSPID, they also considered self-management and a greater involvement of individuals with VSPID in existing activities. The finding that DSPs can contribute to improving the self-management and autonomy of adults with VSPID accords with the research of Hauwert, Meininger, and Kwekkeboom [43], who pointed to the important role of DSPs in adding meaning to different expressions of the self-management of individuals with profound intellectual disabilities.

Another important element of CFP+ is the involvement of family members in the intervention. This involvement is necessary to develop sufficient understanding relating to an individual with VSPID and is a prerequisite for enhancing the individual’s participation, as noted by Axelsson et al. [15]. Moreover, the involvement of the family members of individuals with VSPID enables an exploration of their preferred activities while still living at home. Accordingly, new possibilities may arise. For example, a family member, after remembering that his brother used to enjoy swimming when he lived at home, could try to go swimming with his brother when he visits him at the residential facility.

Because CFP+ is grounded in the definition and operationalization of the concept of participation relating to adults with VSPID, it is tailor-made for this target group. The dimension of visual disabilities was addressed in the DSPs’ worksheets, for example, in the context of searching for new activities that focus on listening to music or experiencing movements. Given that the BPRA is an individually oriented approach and is therefore applicable within multiple contexts, CFP+, which is derived from this approach, could also be suitable for other vulnerable adults who depend on others to express their wishes. However, before attempting to apply CFP+ more broadly, the definition and operationalization of the concept of participation in relation to the target groups must first be established.

### Facilitators and barriers relating to the implementation process

The process evaluation revealed that the implementation of CFP+ was not executed as planned and that the intended dose, reach, and fidelity were not achieved. Nevertheless, DSPs did introduce new activities for adults with VSPID that could be implemented in daily practice, which can be considered a satisfactory outcome of the CFP+ intervention.

Facilitators were evident prior to commencing the CFP+ training program and included, for example, explaining the content of CFP+ to managing staff and convincing them of its added value, establishing arrangements, notably the dates and duration of the training program and the allocation of a classroom within the residential facility to avoid spending extra time and resources on DSPs. An additional facilitator was the association of all of the DSPs with the intended target group. During the training sessions, CFP+ could be adapted to specific opportunities that arose within the residential facility. For example, a number of new activities were developed for adults with VSPID. In general, such facilitators are expected to increase the implementation of an intervention [34, 44].

Although sufficient positive facilitators seemed to exist in advance of the CFP+ implementation, several barriers were also encountered during the process. First, the DSPs perceived their participation to be obligatory; they felt that a top-down decision on the training was being imposed on them. As confirmed by the findings of a study conducted by Knoster, Villa, and Thousand [45], this perception may have negatively influenced their motivation. The trainer, who was used to encountering an open, inquisitive attitude when teaching, experienced a considerable degree of resistance from the DSPs. Second, the training program could not be conducted as planned, which may have resulted in a suboptimal dose.

Nevertheless, CFP+ seemed to have been well understood, and the DSPs worked effectively on goals for the improvement of the participation of adults with VSPID, such as enhancing self-management, developing new roles and activities, and fostering active involvement of these adults in existing activities. However, a surprising finding was that the DSPs did not seem to consider their work on these goals to be an outcome of their engagement with a new intervention; rather, they viewed these efforts as an outcome of other seemingly similar interventions that had been previously introduced and for which they had received training. This may explain why so few DSPs reported using or disseminating CFP+ during the follow-up, and indicated low levels of fidelity and reach that in general may have a negative influence on the implementation of any intervention [34]. Poor concentration during the training sessions, possibly caused by the DSPs’ resistance, was another barrier in the implementation of the CFP+ intervention. However, it is unclear whether the finding that DSPs have not changed their behaviors and attitudes toward participation is only based on their opinion; this can be verified after the effects of the CFP+ intervention have been analyzed.

Another barrier faced in the implementation of the CFP+ intervention, which is supported by Fleuren et al. [35], relates to the DSPs’ perception that they did not have enough time to engage in new activities with the adults with VSPID. In addition, follow-up evaluations could not be conducted with three of the 16 DSPs who received training because they had changed jobs within six months of being trained. A high staff turnover hampers the continuation of an intervention [44] and requires efforts by managers to establish the adoption of interventions such as CFP+ within their facilities.

### Strengths and limitations of the current study

The main strength of this study is that an innovative intervention designed to improve the participation of adults with VSPID, developed by experts in the field of adults with VSPID, was implemented into practice. In addition, the process evaluation enabled the identification and assessment of important barriers and facilitators that can be considered in future implementation exercises once the effectiveness of CFP+ has been validated.

A limitation of this study was that the intervention was only examined in the context of one residential facility, so the results were strongly influenced by the group dynamics of the concerned trainees. It is not clear whether implementation of the intervention in another environment, such as a small-scale facility, would lead to the same results. Consequently, these results cannot be generalized. In addition, CFP+ was tested in a residential facility that differed from the one where the earlier version, CFP, had been tested. Therefore the circumstances under which the intervention was implemented also differed. The results of our previous study [37] showed that the implementation of CFP proceeded smoothly in contrast to the implementation of CFP+. However, the findings of the process evaluation conducted for this study clearly indicated the importance of considering the above-mentioned barriers and facilitators when implementing CFP+ in residential facilities.

### Recommendations for future research and practical implications

The United Nations Convention on the Rights of People with Disabilities obliges governments to invest more in the participation of individuals with disabilities, and this also applies to adults with VSPID. Optimal support for this target group should encompass activities in practice and in policy for advancing optimal participation. As a result, new interventions to improve participation for this target group should be developed and implemented. The level of participation of individuals with VSPID may depend not only on the effectiveness of the intervention itself, but also on whether the implementation has succeeded. In addition, an important facilitator is a government’s willingness to stimulate new policies.

The description of CFP+ presented in this paper opens up opportunities to improve the participation of adults with VSPID. Residential facilities could include CFP+ in their arsenal of methodologies for supporting target populations. When applying CFP+, such facilities should consider the implementation conditions, as indicated by the findings of this study. For example, the manner of recruiting DSPs for the training appeared to prompt their resistance and hampered the intervention’s implementation [34, 45]. Implementation could be encouraged by recruiting early adopters [46], that is, DSPs who recognize and endorse the importance of a new intervention. These early adopters could be identified by the managers of a residential facility prior to implementing CFP+.

Durlak et al. [34] found that the outcomes of an intervention are influenced by its implementation process. Therefore, it is plausible that both facilitators and barriers will influence the effects of CFP+. It is important to determine these effects because despite the suboptimal implementation process observed in this study, the DSPs seemed to have understood and applied some of the tools of the intervention. The findings of an analysis of these effects will be described in subsequent reports.

The conduct of a larger-scale study that includes more residential facilities, DSPs, and adults with VSPID is recommended in order to obtain generalizable findings on the implementation of CFP+. Future studies should also take into account the implementation barriers and facilitators identified in this study and adjust the implementation process in light of the precise contextual factors that contribute to effective implementation [34].

## Conclusion

CFP+, which entails a broad definition and operationalization of the concept of participation that is tailored to adults with VSPID, is aimed at improving the participation of this population [18]. It is an intervention that includes the provision of training for DSPs who work directly with adults with VSPID that is intended to change DSPs’ attitudes toward the participation of such individuals. It also supports them in enhancing the self-management of adults with VSPID and their involvement in existing activities and in developing new daily activities for them.

We have presented the findings of a process evaluation of CFP+ conducted in a residential facility for adults with VSPID. Although some facilitators were present during the CFP+ intervention, the barriers seem to have dominated the implementation process. The most important barrier is likely to have been the DSPs’ experience of overlap with other interventions that they were applying. The fact that they reportedly did not use CFP+ after the training program means that they only applied it during the training period. Nevertheless, the introduction of new activities for adults with VSPID by DSPs, or their enhanced abilities to stimulate greater involvement of these adults in existing activities, may be attributed to the implementation of CFP+.

Future research will focus on examining the effects of CFP+ on the attitudes of DSPs regarding the participation of adults with VSPID and on the actual participation of the target group.

## Supplementary information

**Additional file 1.** Supplementary file with the questions from the online questionnaire, the evaluation forms, and the telephone interview.

## Data Availability

The data analyzed for this study can be made available by the corresponding author on reasonable request.

## Abbreviations

VSPID: Visual and severe or profound intellectual disabilities
DSP: Direct support professional
CFP+: Care for Participation+
CFP: Care for Participation
BPRA: Boston Psychiatric Rehabilitation Approach
PMM: Participation Mind Map

## References

[1] van Splunder J, Stilma JS, Bernsen RMD, Evenhuis HM (2006). Prevalence of visual impairment in adults with intellectual disabilities in the Netherlands: cross-sectional study. Eye..

[2] Limburg H. Epidemiologie van visuele beperkingen en een demografische verkenning. Een studie in opdracht van Stichting InZicht; 2007.

[3] Batshaw ML, Pellegrino L, Roizen LP (2013). Children with disabilities.

[4] Nakken H, Vlaskamp C (2007). A need for a taxonomy for profound intellectual and multiple disabilities. J Policy Pract Intel.

[5] Poppes P, van der Putten AAJ, Vlaskamp C (2010). Frequency and severity of challenging behaviour in people with profound intellectual and multiple disabilities. Res Dev Disabil.

[6] van Timmeren EA, van der Putten AAJ, van Schrojenstein Lantman-de Valk HMJ, van der Schans CP, Waninge A (2016). Prevalence of reported physical health problems in people with severe or profound intellectual and motor disabilities: a cross-sectional study of medical records and care plans. J Intell Disabil Res.

[7] Kiestra T (2005). De unieke handicap, referentiemodel voor meervoudige beperkingen.

[8] Dijkhuizen A, Hilgenkamp TIM, Krijnen WP, van der Schans CP, Waninge A (2016). The impact of visual impairment on the ability to perform activities of daily living in persons with severe/profound intellectual disability. Res Dev Disabil.

[9] Evenhuis HM, Sjoukes L, Koot HM, Kooijman AC (2009). Does visual impairment lead to additional disability in adults with intellectual disabilities?. J Intellect Disabil Res.

[10] Hostyn I, Maes B (2009). Interaction between persons with profound intellectual and multiple disabilities and their partners: a literature review. J Intellect Develop Disabil.

[11] UN General Assembly, Convention on the Rights of Persons with Disabilities, December 2013 2006, A/RES/61/106, Annex I.

[12] Nederlandse overheid. Convention on the rights of persons with disabilities. http://wetten.overheid.nl/BWBV0004045/2016-07-14.Accessed 19 January 2018.

[13] Bigby C, Anderson S, Cameron N. Identifying conceptualizations and theories of change embedded in interventions to facilitate community participation for people with intellectual disability: a scoping review. J Appl Res Intellect Disabil. 2018;31(2):165–80. 10.1111/jar.12390 28799696

[14] Whiteneck G, Dijkers MP (2009). Difficult to measure constructs: conceptual and methodological issues concerning participation and environmental factors. Arch Phys Med Rehab.

[15] Axelsson AK, Imms C, Wilder J (2014). Strategies that facilitate participation in family activities of children and adolescents with profound intellectual and multiple disabilities: parents’ and personal assistants’ experiences. Disabil Rehabil.

[16] Boren T, Granlund M, Wilder J, Axelsson AK (2016). Sweden’s LSS and social integration: an exploration of the relationship between personal assistant type, activities, and participation for children with PIMD. J Policy Pract Intel..

[17] Schalock RL, Brown I, Brown R, Cummins RA, Felce D, Matikka L (2002). Conceptualization, measurement, and application of quality of life for persons with intellectual disabilities: report of an international panel of experts. Ment Retard.

[18] Hanzen G, van Nispen RMA, van der Putten AAJ, Waninge A (2017). Participation of adults with visual and severe or profound intellectual disabilities: definition and operationalization. Res Dev Disabil.

[19] Hanzen G, Waninge A, Vlaskamp C, van Nispen RMA, van der Putten AAJ (2018). Participation of adults with visual and severe or profound intellectual disabilities: analysis of individual support plans. Res Dev Disabil.

[20] Talman L, Gustafsson C, Stier J, Wilder J. Staffs’ documentation of participation for adults with profound intellectual disability or profound intellectual and multiple disabilities. Disabil Rehabil. 2017:1464–5165. 10.1080/09638288.2017.1340979. 10.1080/09638288.2017.1340979 28633543

[21] Wolfensberger W (2000). A brief overview of social role valorization. Ment Retard.

[22] Bigby C, Clement T, Mansell J, Beadle-Brown J (2009). ‘It’s pretty hard with our ones, they can’t talk, the more able bodied can participate’: staff attitudes about the applicability of disability policies to people with severe and profound intellectual disabilities. J Intellect Disabil Res.

[23] Talman L, Wilder J, Stier J, Gustafsson C (2019). Staff members and managers' views of the conditions for the participation of adults with profound intellectual and multiple disabilities. J Appl Res Intellect..

[24] Maxwell G, Alves I, Granlund M (2012). Participation and environmental aspects in education and the ICF and the ICF-CY: findings from a systematic literature review. Dev Neurorehabil.

[25] Kröber H, Verdonschot M (2012). Professionals en inclusieve praktijken. Nederlands tijdschrift voor de zorg aan mensen met verstandelijke beperkingen (NTZ)..

[26] McConkey R, Collins S (2010). The role of support staff in promoting the social inclusion of persons with an intellectual disability. J Intellect Disabil Res.

[27] Bolsenbroek A. (2014). De VeranderKIZT voor Zeggenschap en Inclusie*.* https://www.inclusionlab.nl/. Accessed 20 April 2019.

[28] Kruijswijk W, Veer M van der, Brink C, Calis W, Maat J van de, Redeker I. Aan de slag met sociale netwerken. Movisie, Vilans; 2014.

[29] Sandjojo J, Zedlitz AMEE, Gebhardt WA, Hoekman J, Dusseldorp E, den Haan JA (2018). Training staff to promote self-management in people with intellectual disabilities. J Appl Res Intellect.

[30] Mansell J, Beadle-Brown J (2012). Active support: enabling and empowering people with intellectual disabilities.

[31] Stancliffe RJ, Jones E, Mansell J, Lowe K (2008). Active support: a critical review and commentary. J Intellect Develop Disabil.

[32] Dröes J (1992). Een opleidingsprogramma voor rehabilitatie bij chronisch psychiatrisch problematiek.

[33] Anthony WA, Cohen MR, Farkas MD, Gagne C (2002). Psychiatric rehabilitation.

[34] Durlak JA, DuPre EP (2008). Implementation matters: a review of research on the influence of implementation on program outcomes and factors affecting implementation. Am J Commun Psychol.

[35] Fleuren MAH, Paulussen TG, WM, van Dommelen P, van Buuren S (2014). Towards a measurement instrument for determinants of innovations. Int J Qual Health C.

[36] Moore GF, Audrey S, Barker M, Bond L, Bonell C, Hardeman W (2015). Process evaluation of complex interventions: Medical Research Council guidance. BMJ..

[37] Hanzen G, Korevaar EL, van der Putten AAJ, Zijlstra A, Waninge A (2016). Zorg voor Participatie: kwalitatief onderzoek naar de toepasbaarheid en resultaten van een nieuwe methodiek om de participatie te vergroten van volwassenen met visuele en (zeer) ernstige verstandelijke beperkingen. Nederlands tijdschrift voor de zorg aan mensen met verstandelijke beperkingen (NTZ).

[38] Pickens J, Borkowski N (2005). Attitudes and perceptions. Organizational behaviour.

[39] Korevaar L, Dröes J (2016). Handboek Rehabilitatie voor zorg en welzijn.

[40] Swildens W, van Busschbach J, Michon H, Kroon H, Koeter M, Wiersma D (2011). Effectively working on rehabilitation goals: 24-month outcome of a randomized controlled trial of the Boston psychiatric rehabilitation approach. Can J Psychiatr.

[41] ICD-10 Version:2016. http://apps.who.int/classifications/icd10/browse/2016/en#/H54 Accesed 30 july 2018.

[42] Moore, G. e. a. (2017). Process evaluation of complex interventions. UK medical research council (MRC) guidance. https://www.Mrc.ac.uk/documents/pdf/mrc-phsrn-process-evaluation-guidance-final. Accessed 18 May 2019.

[43] Hauwert SAC, Meininger HP, Kwekkeboom MH (2014). Eigen regie in (dag)rapportages over mensen met ernstige meervoudige beperkingen. Een discoursanalyse. Nederlands Tijdschrift voor de zorg aan mensen met verstandelijke beperkingen (NTZ).

[44] Elinder LS, Sundblom E, Zeebari Z, Bergström H (2018). Effect and process evaluation of a structural health intervention in community residences for adults with intellectual disabilities. J Policy Pract Intel.

[45] Knoster T, Villa R, Thousand J, Villa R, Thousands J (2000). A framework for thinking about systems change. Restructuring for caring and effective education: Piecing the puzzle together.

[46] Rogers E (2003). Diffusion of innovations 5^th^ ed.

