# Supplementary material for: Improving the participation of adults with visual and severe or profound intellectual disabilities: a process evaluation of a new intervention
Source: BMC Health Serv Res. 2020 Apr 16;20:319. doi: 10.1186/s12913-020-05161-1 (PMC7164344; doi:10.1186/s12913-020-05161-1)
Supplement: Supplementary file 1 — Additional file 1. Supplementary file with the questions from the online questionnaire, the evaluation forms, and the telephone interview. [file 12913_2020_5161_MOESM1_ESM.docx]

**Supplementary file with the questions from the online questionnaire, the evaluation forms, and the telephone interview.**

Online questionnaire completed by Direct support professionals (DSPs)

What is your age?

What is your gender?

What is your position? (support professional in a home group, support professional in a day-time activity group, or support professional with an additional coordinating role)

How much time did you spend using the CFP+ methodology during the six-month period that followed the training?

Have you applied the exercises in the worksheets of the CFP+ during the post-training period in relation to the adult with VSPID with whom you were associated during the study?

Evaluation forms completed by DSPs after the training CFP+:

*The questions were answered on a five-point Likert scale (from very good to very bad) with room for explanation*

What is your opinion about the didactic qualities of the trainer?

What is your opinion about the applicability of the teaching material?

What is your opinion about the relevance of the training for your work situation?

What is your opinion about the making of the homework assignments between the different meetings of the training?

What is your opinion about the other participants in the training?

*The following questions were not scored on a Likert scale:*

What is your overall impression of the training?

Do you have any other comments on the training?

Telephone interview conducted with DSPs, 6 months after the training

Have you applied CFP+ to enhance the self-management of adults with VSPID?

Have you applied CFP+ to explore new roles for adults with VSPID?

Have you applied CFP+ to develop new activities for adults with VSPID?

Have you applied CFP+ to increase the active involvement in existing activities of adults with VSPID?

Did you inform your team members about CFP+ after the training?
